# Monitoring Italian COVID-19 spread by a forced SEIRD model

**DOI:** 10.1371/journal.pone.0237417

**Published:** 2020-08-06

**Authors:** Elena Loli Piccolomini, Fabiana Zama

**Affiliations:** 1 Department of Computer Science and Engineering, University of Bologna, Bologna, Italy; 2 Department of Mathematics, University of Bologna, Bologna, Italy; Consejo Superior de Investigaciones Cientificas (CSIC), SPAIN

## Abstract

Due to the recent evolution of the COVID-19 outbreak, the scientific community is making efforts to analyse models for understanding the present situation and for predicting future scenarios. In this paper, we propose a forced Susceptible-Exposed-Infected-Recovered-Dead (fSEIRD) differential model for the analysis and forecast of the COVID-19 spread in Italian regions, using the data from the Italian Protezione Civile (Italian Civil Protection Department) from 24/02/2020. In this study, we investigate an adaptation of fSEIRD by proposing two different piecewise time-dependent infection rate functions to fit the current epidemic data affected by progressive movement restriction policies put in place by the Italian government. The proposed models are flexible and can be quickly adapted to monitor various epidemic scenarios. Results on the regions of Lombardia and Emilia-Romagna confirm that the proposed models fit the data very accurately and make reliable predictions.

## Introduction

The recent evolution of the COVID-19 epidemic has renewed the interest of the scientific and political community in the mathematical models for the epidemic. Many researchers all over the world are proposing new refined models to analyse the present situation and predict possible future scenarios (see for example [1–6]).

With this paper, we hope to contribute to the ongoing research on this topic and to give a practical instrument for a deeper comprehension of the outbreak evolution.

The modelling of epidemics is currently performed by Ordinary Differential Equations (ODEs) deterministic compartmental models [7, 8], or by stochastic procedures [9]. We consider here deterministic compartmental models, based on a system of initial value ODEs, whose theory has been studied since the beginning of the century by W.O. Kermack and A. G. MacKendrick [10] who proposed the basic Susceptible-Infected-Removed (SIR) model. Since then, many modifications have been developed to study the epidemics of different infectious diseases [7]. These models split the population into groups, compartments, and reproduce their behaviour by formalising their reciprocal interactions. For example, the SIR model groups are: susceptible, who can catch the disease, infected, who have the disease and can spread it, and removed, who have either had the disease or have recovered, are immune or isolated until recovery. The Susceptible-Exposed-Infected-Removed (SEIR) model also considers the exposed group, containing individuals who are in the incubation period. Since we believe that relevant information concerns not only infected but also Recovered and Dead populations, we choose to split removed population into Recovered and Dead, obtaining the SEIRD model. Such an approach is similar to [11], without accounting for infections from deceased to susceptibles, that do not apply to COVID-19.

Compared to previous outbreaks, such as SARS-CoV or MERS-CoV [2], the COVID-19 epidemic had a more rapid spread and it was proclaimed pandemic by the WHO on 11/03/2020. Indeed, the number of infected people would grow exponentially, if not contained by social isolation of the affected areas, as first evidenced by the COVID-19 outbreak in the Chinese city of Wuhan in December 2019 and currently applied almost worldwide.

In particular, the Italian government has started to impose severe restrictions since 09/03/2020 registering a substantial reduction in the growth rate of infected people ever since. The introduction of such social restricting measures requires an adaptation of the standard epidemic models to this new situation.

The evolution of the infected population depends on the basic reproduction number, denoted as *R*_0_, which measures how transferable a disease is. This quantity determines whether the infection will spread exponentially (*R*_0_ > 1), die out (*R*_0_ < 1), or remain constant (*R*_0_ = 1). In this paper we propose a time-dependent infection rate function *R*_0_(*t*), instead of a constant parameter, since we believe that it could provide a model that better represents the COVID-19 outbreak evolution in Italy. The idea of introducing a non-constant infection rate has been adopted in several different situations. See for example in [7], ch. 5, where periodic infection rate functions model influenza epidemic seasonality, naming such modified models as *forced models*. In [12], an exponential infection rate function was used to represent the Ebola outbreak. Recently, in [13, 14] the authors have proposed multi-scale models with several time-dependent parameters, to study COVID-19 epidemic.

In this paper, we propose two infection rate piecewise functions and we calibrate the two forced SEIRD models employing the COVID-19 Italian data, relative to Lombardia and Emilia-Romagna regions. The actual data is relative to about three months, where the peak of the infected population has already been reached. The calibrated models yield excellent data fit on both regions. Moreover, we have simulated a prevision based on early-stage epidemic data, relative to the first 30 days: the comparison of the results with the real epidemic evolution shows a difference of very few days between the real and predicted peaks.

## Materials and methods

In this section we introduce the epidemic data used for our analysis of COVID-19 in Italy, we present the proposed mathematical model, the method used for calibrating the parameters and the strategies applied for predictions.

### Epidemic data and containment measures in Italy

In our analysis we refer to the dataset of the Italian Civil Protection Department, which is described in [15] and publicly available in the GitHub repository [16]. The data have been collected since 24/02/2020. We consider the infected population *I* as the infected active cases (field name: totale_attualmente_positivi in Table 1 [15]). The Recovered *R* and Dead *D* compartments are given in the fields dimessi_guariti and deceduti respectively (Table 1 [15]). This study considers two Italian regions, Lombardia and Emilia-Romagna.

On 09/03/2020 lockdown was declared for the entire country, while more severe restrictions were adopted in the different regions. For example in Lombardia, the Codogno municipal area was locked down from 21/02/2020 up to 08/03/2020, conversely in Emilia-Romagna the complete closure of the Medicina municipal area was applied from 16/03/2020 up to 04/04/2020 [17]. Therefore, we have chosen to calibrate the models in each region separately.

Further information about COVID-19 in Italy can be found at [18].

### The proposed forced SEIRD model

The epidemiological compartmental models divide the population into groups, whose evolution in time is described by continuous functions, and describe the relations between the groups with ODEs. In this paper we use a SEIRD model [7, 11], which considers five population compartments: susceptible (S), exposed (E), infected (I), Recovered (R) and Dead (D). The system of equations in the SEIRD model is given by:

$$\begin{array}{cc} & \frac{dS}{dt}=-\frac{\beta }{N}SI\\ & \frac{dE}{dt}=\frac{\beta }{N}SI-\alpha E\\ & \frac{dI}{dt}=\alpha E-\frac{1}{T_{I}}I\\ & \frac{dR}{dt}=\frac{\left(1-f\right)}{T_{I}}I\\ & \frac{dD}{dt}=\frac{f}{T_{I}}I \end{array}$$ (1)

where *N* is the total population, i.e. *N* = *S* + *E* + *I* + *R* + *D* at each time *t*, *β* is the infection rate, i.e. a coefficient accounting for the susceptible people get infected by infectious people, *α* is the incubation rate for the transition from exposed to infected, *T*_*I*_ is the average infectious period and *f* is the fraction of all the removed individuals who die. The basic reproduction number *R*_0_ has the following expression:

$$\begin{array}{c} R_{0}=\beta T_{I}. \end{array}$$ (2)

The system (1) is solved by starting from an initial time *t* = *t*_0_ where the values of the populations *S*(*t*_0_), *E*(*t*_0_), *I*(*t*_0_), *R*(*t*_0_), *D*(*t*_0_) are assigned on the basis of the available data and integrated up to a final time *T*.

However, the SEIRD model (1) with constant parameters *β*, *α*, *f* does not fit well the available data for more than few days, due to the rapidly changing social scenarios during the initial period of the COVID-19 spread in Italy. In particular, since the applied restrictions, described in the previous section, cause a decrease in the number of contacts between infected and susceptible, we model the coefficient *β* in (1) as a time-dependent decreasing function *β*(*t*), yielding a *forced* SEIRD model fSEIRD [7].

Moreover, to improve the model flexibility, we split the integration interval [*t*_0_, *T*] into *p* sub-intervals [*t*_*k*_, *t*_*k*+1_], *k* = 1, … *p* and propose two alternative piecewise infection rate functions: a rational function *β*_*r*_(*t*) or an exponential function *β*_*e*_(*t*). In each sub-interval [*t*_*k*_, *t*_*k*+1_] the infection rate functions have the following expression:

$$\begin{array}{cc} & \beta_{r}\left(t\right)=\beta \left(t_{k}\right)(1-\rho_{k}\left(t-t_{k}\right)/t))\;\;t\in \left(t_{k},t_{k+1}\right],\;\;\rho_{k}\in \left(0,1\right) \end{array}$$ (3)

$$\begin{array}{cc} & \beta_{e}\left(t\right)=\beta \left(t_{k}\right)e^{-\rho_{k}\left(t-t_{k}\right)},\;\;t\in \left(t_{k},t_{k+1}\right],\;\;\rho_{k}\geq 0. \end{array}$$ (4)

for an assigned starting value *β*(*t*_0_). The parameters *α* and *f* are assumed to be constant on each sub-interval, hence they are represented by piecewise constant functions:

$$\begin{array}{c} \begin{array}{cc} \alpha \left(t\right)=\alpha_{k}, & \alpha_{k}\geq 0\\ f\left(t\right)=f_{k}, & f_{k}\geq 0 \end{array},\;t\in \left(t_{k},t_{k+1}\right],\;k\geq 0. \end{array}$$ (5)

The proposed fSEIRD model is expressed as follows:

$$\begin{array}{cc} & \frac{dS}{dt}=-\frac{\beta (t)}{N}SI\\ & \frac{dE}{dt}=\frac{\beta (t)}{N}SI-\alpha \left(t\right)E\\ & \frac{dI}{dt}=\alpha \left(t\right)E-\frac{1}{T_{I}}I\\ & \frac{dR}{dt}=\frac{\left(1-f(t)\right)}{T_{I}}I\\ & \frac{dD}{dt}=\frac{f(t)}{T_{I}}I \end{array}$$ (6)

with the following time dependent basic reproduction function:

$$\begin{array}{c} R\left(t\right)=\beta \left(t\right)T_{I}. \end{array}$$ (7)

In the following, we name fSEIRDr and fSEIRDe the model (6) with the rational infection rate (3) and the exponential infection rate (4), respectively.

### Parameter estimation and prevision

In order to estimate the parameters *α*_*k*_, *f*_*k*_ and *ρ*_*k*_ in (3), (4) and (5), we fit the solution of (6) to the measured data of the infected, recovered and Dead populations $\hat{I},\hat{R},\hat{D}$, relative to *M* days starting from 24/02/2020. We calibrate the parameters of fSEIRDr or fSEIRDe by solving a nonlinear least-squares problem with positivity and bound constraints. Mathematically, the problem can be formulated as follows. Let **u**(*t*) be the multi-value function:

$$\begin{array}{c} \text{u}\left(t\right):\left[t_{0},T\right]⟶{\text{R}}^{5},\;\;u\left(t\right)=\left(S\left(t\right),E\left(t\right),I\left(t\right),R\left(t\right),D\left(t\right)\right), \end{array}$$

solution of the ODE system (6) and let

$$\begin{array}{c} \text{a}=\left(\alpha_{1},\ldots ,\alpha_{p}\right),\;\text{f}=\left(f_{1},\ldots ,f_{p}\right),\;\text{r}=\left(\rho_{1},\ldots ,\rho_{p}\right) \end{array}$$

be the vectors of the model parameters. The function **u**(*t*) depends on the unknown parameters **a**, **f**, **r**, hence we write **u**(*t*; **a**, **f**, **r**) ≡ **u**(*t*). We define the restriction of **u**(*t*; **a**, **f**, **r**) to the three measured populations (*I*(*t*), *R*(*t*), *D*(*t*)) by means of the function:

$$\begin{array}{c} \text{v}\left(t;\text{a},\text{f},\text{r}\right):\left[t_{0},T\right]⟶{\text{R}}^{3},\;\;v\left(t,\text{a},\text{f},\text{r}\right)=\left(I\left(t\right),R\left(t\right),D\left(t\right)\right). \end{array}$$

For each day *t*(*i*) in the vector of times **t** = (*t*(0), *t*(1), … *t*(*M*)), we compute the vectors **v**(*t*(*i*);**a**, **f**, **r**) ∈ **R**^3^, *i* = 1, … *M*, and we stack them into the vector **v**(**t**;**a**, **f**, **r**), of length 3 ⋅ *M*. Analogously, we define the observations vector at time *t*(*i*) as $\text{y}(i)=(\hat{I}(i),\hat{R}(i),\hat{D}(i))\in {\text{R}}^{3},$, and we stack the vectors **y**(*i*) *i* = 1, … *M* into the vector **Y** of length 3 ⋅ *M*. The model parameters **a**, **f**, **r** are estimated by solving the following nonlinear constrained least-squares problem:

$$\begin{array}{c} \underset{\text{a},\text{f},\text{r}}{\text{min}}{\left\|\text{v}\left(\text{t};\text{a},\text{f},\text{r}\right)-\text{Y}\right\|}_{2}^{2}\;\;s.t.\;\text{a}\geq 0,\;\text{f}\geq 0,\;\text{r}\geq 0. \end{array}$$ (8)

Conceptually, this least-squares optimization is equivalent to a maximum likelihood estimation, where the likelihood of data given parameters is a multivariate normal distribution with mode **v** and spherical unit covariance. Eq (8) may be interpreted as the minimum of the negative log of this likelihood. The iterative trust-region based method implemented by the lsqnonlin Matlab function is applied to solve problem (8) (see [19] for details about this aspect). It is well known that the nonlinear problem (8) may have many local minima and that the iterative method, implemented by the solver, converges to one of them. Furthermore, the starting guess is discriminatory for the accuracy of the computed solution. To choose suitable starting guesses approximating the real parameters, we perform a two phase process, where in phase 1 we estimate the parameters *β*, *α*, *f* of the classical SEIRD model (1) on a restricted number of days *M*_*l*_ < *M* [20] and then, in phase 2, we calibrate the parameters of fSEIRDr or fSEIRDe by applying the solutions of phase 1 as starting guesses. Indeed, the identification process in phase 2 requires initial values *α*_0_, *f*_0_, *ρ*_0_ for the iterative process solving (8) and *β*(*t*_0_) for the computation of the functions *β*(*t*) (3) or (4), therefore the parameters *α*, *f*, *β* computed in phase 1 are assigned as starting guesses. The starting value *ρ*_0_ is fixed as *ρ*_0_ = 0.9. To define the intervals [*t*_*k*_, *t*_*k*+1_] in Eqs (3) and (4), we fix a value Δ_*t*_ > 0 and partition the measurements interval [*t*(0), *T*] in *p* sub-intervals [*t*_*k*_, *t*_*k*+1_] where

$$\begin{array}{c} t_{0}\equiv t\left(0\right)<t_{1}<t_{2}<\cdots <t_{p}\equiv T,\;\;\text{and}\;t_{k+1}=t_{k}+\Delta_{t},\;\;k<p-1 \end{array}$$ (9)

allowing the length of last interval [*t*_*p*−1_, *t*_*p*_] to be larger than Δ_*t*_ > 0. Finally, we apply (8) to compute the parameters **a**, **f**, **r**.

After having carried out the estimation of the model parameters we use fSEIRDr, fSEIRDe to monitor the epidemic evolution and make some predictions about the infection behaviour in the successive few weeks. This information is extremely important to predict the length of the epidemic spread.

## Results

In this section, we test the fSEIRDe and fSEIRDr models using data of Lombardia and Emilia-Romagna regions. In paragraph Model Calibration, we calibrate the two models on the whole time interval available in the dataset from 24/02/2020 to 24/05/2020, which includes the epidemic Infections peak. Then in paragraph Epidemic Evolution Forecast we test the models behaviour restricting the calibration time to the interval [24/02/2020, 27/03/2020], to monitor the COVID-19 evolution and forecast of the epidemic peak.

The differential systems are solved applying the ode45 Matlab function, implementing a variable step Runge-Kutta method based on Dormand-Prince formulae, with the following initial conditions: $I(t_{0})=\hat{I}(1)$, $R(t_{0})=\hat{R}(1)$, $D(t_{0})=\hat{D}(1)$ where $\hat{I}(1),\hat{R}(1),\hat{D}(1)$ correspond to the infected, recovered, Dead individuals in the first measurement day. Concerning the exposed population, for which no measurement is available, we set *E*(0) = 10 ⋅ *I*(*t*_0_) in Emilia-Romagna. This value is reasonable for the initial epidemic evolution, leading to a basic reproduction index *R*_0_ ≃ 6.6. Concerning the region of Lombardia, the same initial value *E*(0) = 10 ⋅ *I*(*t*_0_) leads to an excessively high reproduction index. However, since the available data in the first days of the outbreak diffusion were uncomplete, we have decided to use that value of *E*(0) and concentrate our analysis in the subsequent times of the pandemic (from day 20th onwards). In future software versions, the value of *E*(0) could be possibly estimated from data.

Finally we set *S*(*t*_0_) = *N* − *E*(*t*_0_) − *I*(*t*_0_) − *R*(*t*_0_) − *D*(*t*_0_) where and *N* is the total population of the region.

To evaluate the estimated data, we consider the Root Mean Squared Error (RMSE):

$$\begin{array}{c} RMSE=\sqrt{MSE},\;MSE=\frac{\sum_{i}^{N_{c}}{\left(X_{mod}\left(i\right)-X_{data}\left(i\right)\right)}^{2}}{N_{c}} \end{array}$$ (10)

where *X*_*mod*_ represents a single population among the modelled compartments ({I,R,D} respectively) and *X*_*data*_ is the corresponding compartment data in the calibration days *t*(*i*), *i* = 1, 2, …, *N*_*c*_, *N*_*c*_ ≤ *M*, and the Bayesian Information Criterion (BIC) [21], defined as follows:

$$\begin{array}{c} BIC=N_{p}\;\text{log}\left(N_{c}\right)+\text{log}\left(MSE\right) \end{array}$$ (11)

where *N*_*p*_ represents the number of the parameters estimated by the model. The RMSE measures the average error performed by the model in predicting the outcome for an observation while the BIC takes into account the number of model estimated parameters and tends to penalize the inclusion of additional parameters. In both cases the best models are given by the lowest values.

In this study we choose as the average infectious period *T*_*I*_ = 20(*d*), the time in which the viral RNA may be detected by means of laboratory procedures, as reported in [3]. This value is different from the infectious period reported in [22] which is variable in the interval [2(*d*), 14(*d*)] with possible outliers at 24(*d*) and 27(*d*).

All the computations are performed using Matlab R2020a 2,9 GHz Intel Core i7 quad-core 16 GB ram.

### Model calibration

**Fig 1:**
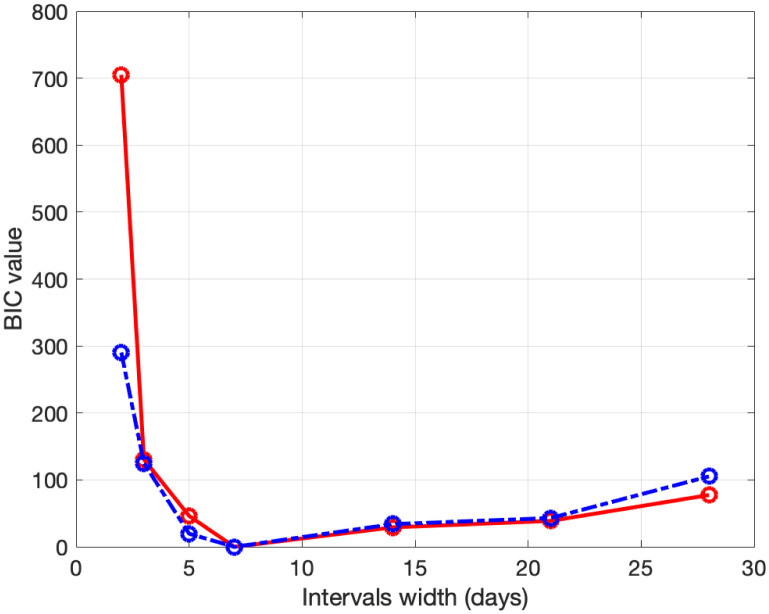
BIC parameters for Emilia-Romagna. Scaled BIC parameters Δ(*BIC*)_*i*_ (12) computed for intervals widths {3, 5, 7, 14, 21, 28} with fSEIRDe (red continuous line), fSEIRDr (blue dash-dotted line) models.

**Fig 2:**
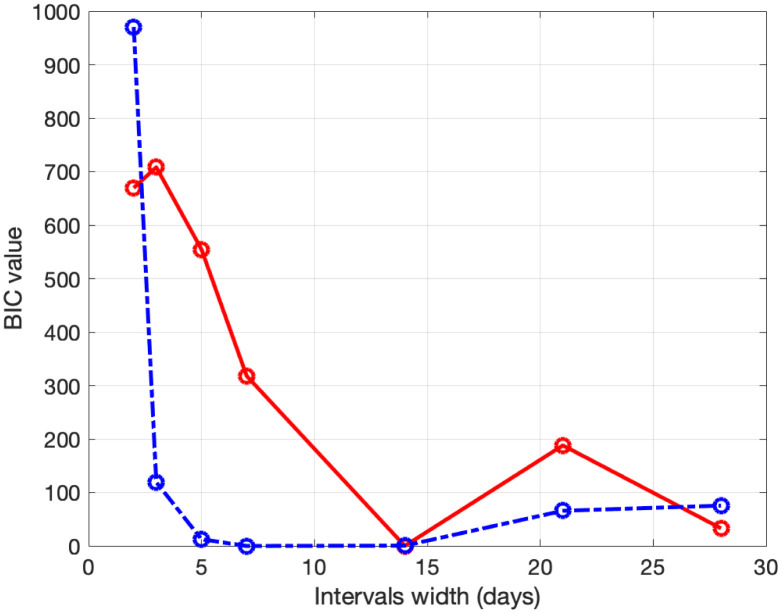
BIC parameters for Lombardia. Scaled BIC parameters Δ(*BIC*)_*i*_ (12) computed for intervals widths {3, 5, 7, 14, 21, 28} with fSEIRDe (red continuous line), fSEIRDr (blue dash-dotted line) models.

**Fig 3:**
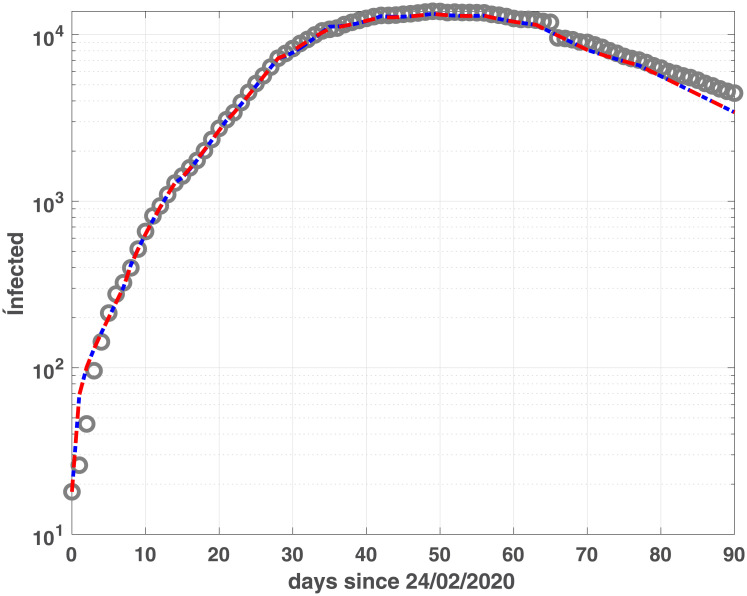
Infected compartments for Emilia-Romagna. Infected data (gray circles) and infected modelled population, obtained by fSEIRDr (blue dash-dotted line) and fSEIDRe (red continuous line).

**Fig 4:**
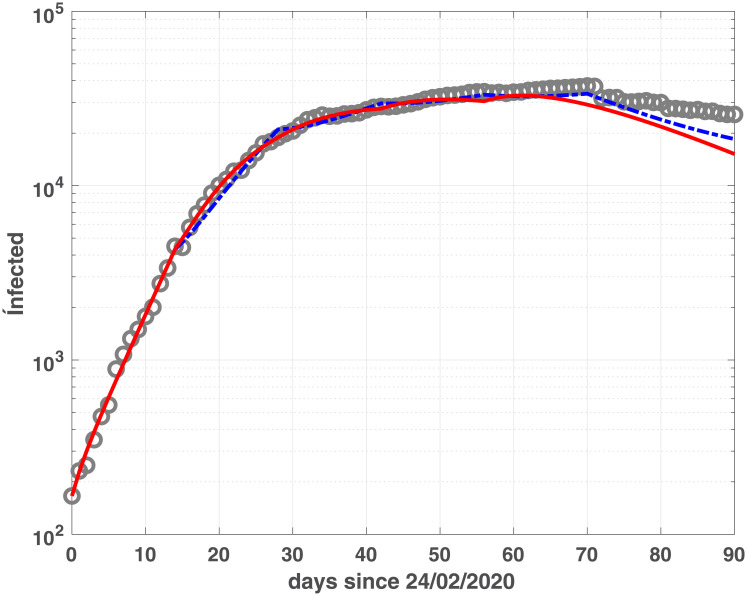
Infected compartments for Lombardia. Infected data (gray circles) and infected modelled population, obtained by fSEIRDr (blue dash-dotted line) and fSEIDRe (red continuous line) for Lombardia.

To calibrate fSEIRDr and fSEIRDe models we perform the phase 1 to obtain the starting guesses to be used in phase 2. We apply the SEIRD model (1), with initial parameters *α* = 0.1 *d*^−1^, *f* = 0.1 and *β* = 0.25 *d*^−1^, on the data [16] relative to the first 15 days [24/02/2020, 09/03/2020] for both regions. The estimated parameters, applied as starting guesses in phase 2, are reported in Table 1. Concerning phase 2, we calibrate the fSEIRDr or fSEIRDs parameters using the available data relative of the first 90 days from 24/02/2020 up to 24/05/2020 and set *ρ*_0_ = 0.9. We first investigate the choice of time partitions (9) by changing the value of the intervals length Δ_*t*_. Such value should balance the increasing number of parameters, when Δ_*t*_ is small, with the increasing value of the RMSE, for large Δ_*t*_ values. This behaviour has been studied by computing the *BIC*_*i*_ values corresponding to each $\Delta_{t}\in ℐ$, where ℐ≡{3,5,7,14,21,28} days and then computing the values of the scaled BIC parameter [23]:

$$\begin{array}{c} \Delta {\left(BIC\right)}_{i}=BIC_{i}-BIC_{min},\;i=1,\ldots ,Ik \end{array}$$ (12)

where *BIC*_*min*_ is the minimum value of the *BIC*_*i*_ and *Ik* is the number of elements in the set ℐ; the best value is obtained when Δ(*BIC*)_*i*_ = 0. Comparing the plots reported in Figs 1 and 2, we observe that the two models reach the minimum BIC when Δ_*t*_ is 7 for Emilia-Romagna data. For Lombardia we observe that fSEIRdr has very small values for 3 ≤ Δ_*t*_ ≤ 14 while fSEIRDe is very sensitive to Δ_*t*_ and reaches its minimum when Δ_*t*_ = 14. Therefore, in the following we use Δ_*t*_ = 14 for Lombardia and Δ_*t*_ = 7 for Emilia-Romagna in the calibration of the parameters and we show in Figs 3 and 4 the infected populations obtained by both models in Emilia-Romagna and Lombardia respectively.

**Table 1:** Phase 1 SEIRD parameters.

Region	*α*_0_ (*d*^−1^)	*f*_0_ (−)	*β*_0_ (*d*^−1^)
Lombardia	4.09 10^−2^	0.27	1.665
Emilia R.	0.258	0.567	0.457

Parameters obtained in phase 1 by SEIRD (1) in the first 15 days measurements: [24/02/2020, 09/03/2020].

**Fig 5:**
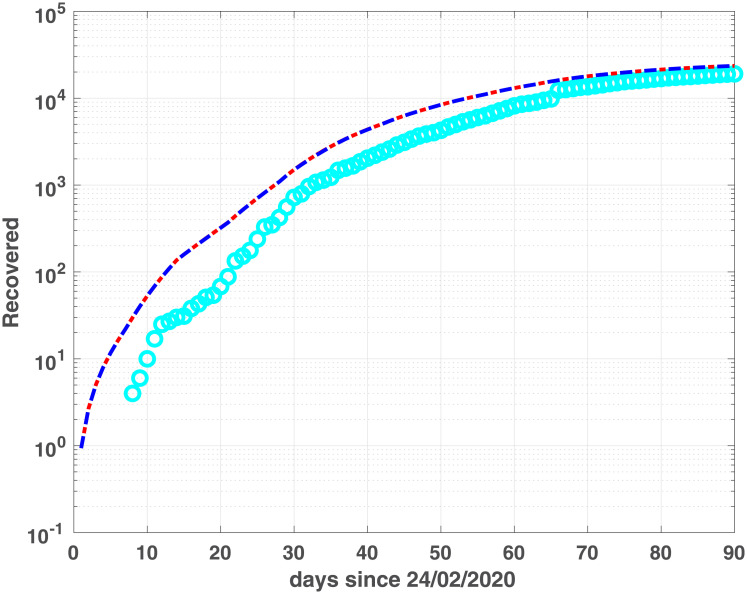
Recovered compartments for Emilia-Romagna. Recovered data (cyan dots) and Recovered modelled population, obtained by fSEIRDr (blue dash-dotted line) and fSEIDRe (red continuous line).

**Fig 6:**
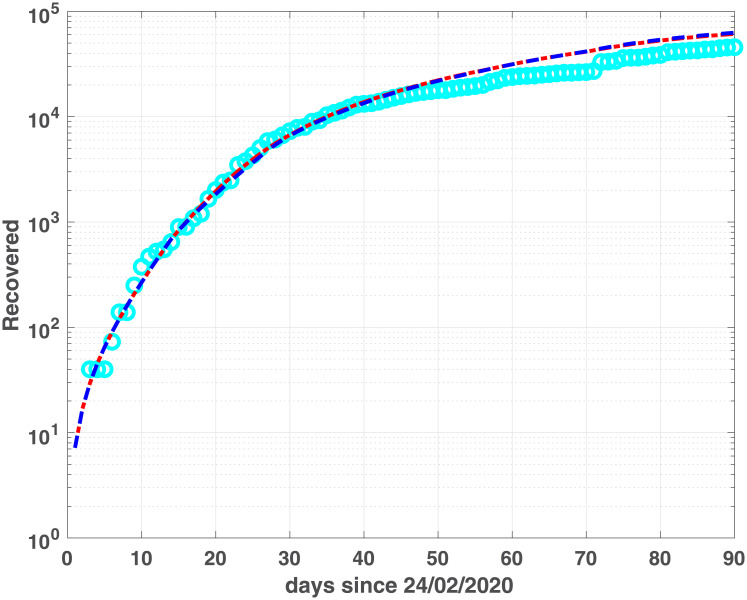
Recovered compartments for Lombardia. Recovered data (cyan dots) and Recovered modelled population, obtained by fSEIRDr (blue dash-dotted line) and fSEIDRe (red dashed line).

Regarding Lombardia region, we observe a quite good fit of the Recovered population (Fig 6) while the Dead population is well fitted in the first 40 days, successively the model tends to over estimate the data (Fig 8). Conversely, for Emilia-Romagna data, the good fit of the infected population does not extend to Recovered and Dead compartments, although the Recovered data are quite well fitted in the last 10 days (Fig 5).

**Fig 7:**
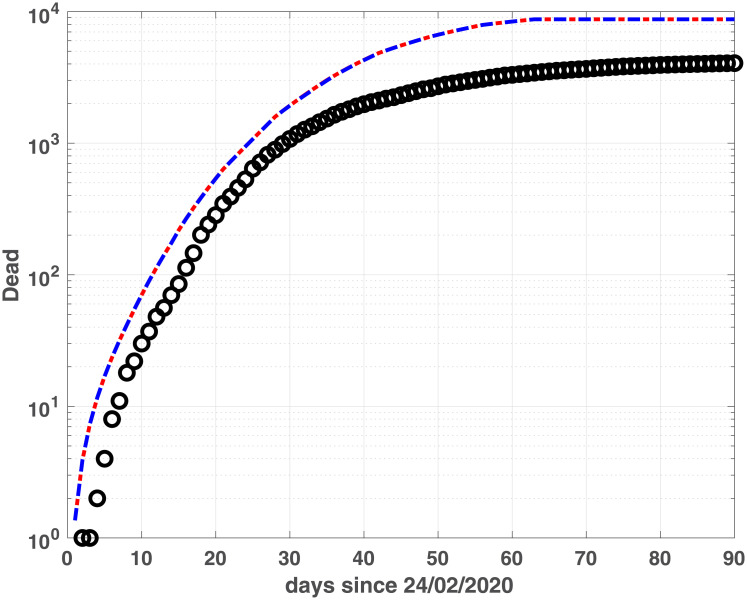
Recovered compartments for Emilia-Romagna. Dead data (black dots) and Dead modelled population, obtained by fSEIRDr (blue dash-dotted line) and fSEIDRe (red dashed line).

**Fig 8:**
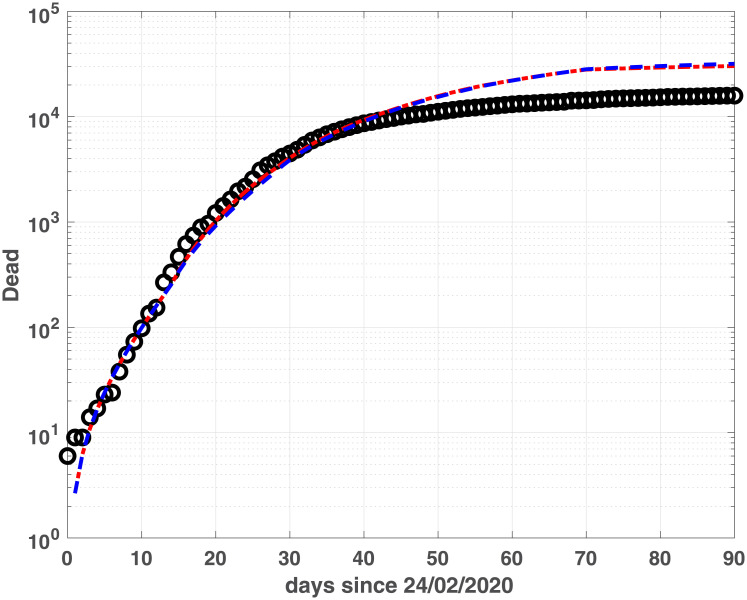
Recovered compartments for Lombardia. Dead data (black dots) and Dead modelled population, obtained by fSEIRDr (blue dash-dotted line) and fSEIDRe (red dashed line).

**Fig 9:**
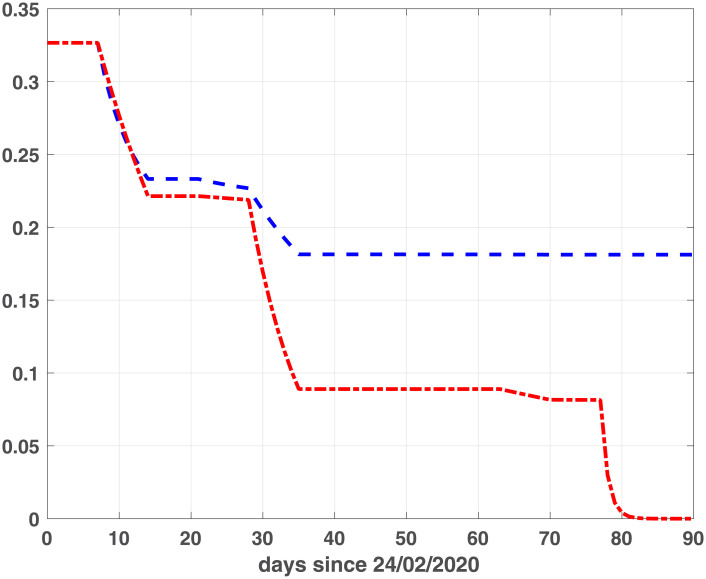
Infection rate in Emilia-Romagna. Functions *β*_*r*_(*t*) (blue dashed line), *β*_*e*_(*t*) (red dash-dotted line), obtained by fSEIRDr and fSEIDRe respectively.

**Fig 10:**
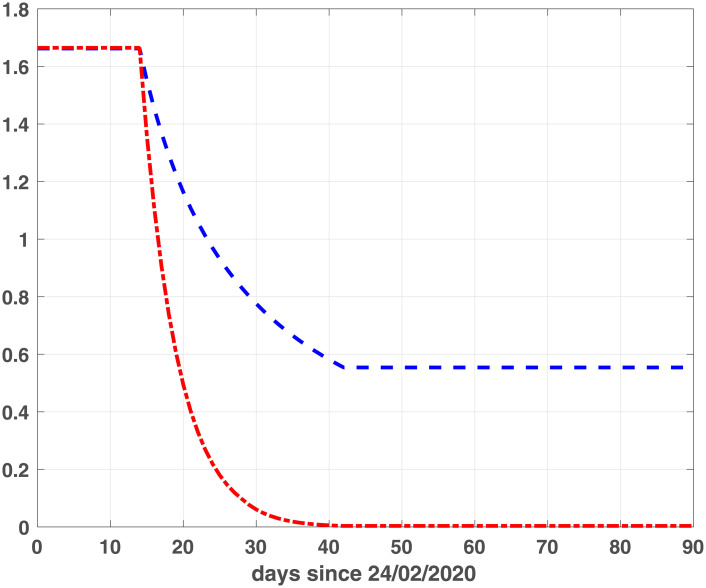
Infection rate in Lombardia. Infection rate functions *β*_*r*_(*t*) (blue dashed line), *β*_*e*_(*t*) (red dash-dotted line), obtained by fSEIRDr and fSEIDRe respectively.

The two models show similar fitting properties, as confirmed by the BIC and RMSE values in Table 2 as well as by the infected, (Figs 3 and 4), Recovered (Figs 5 and 6) and Dead (Figs 7 and 8) populations. Finally, we observe in Figs 9 and 10 the different behaviour of the infection rate function *β*(*t*) in the two considered regions. The exponential function *β*_*e*_ has a steeper decreasing behaviour, compared to the rational function *β*_*r*_. Hence we expect that fSEIRDe forecasts a reduction of the epidemic spread in a shorter time than fSEIRDr. Conditioned upon this model space, from Table 2 there is strong evidence in favour of the fSEIRDr model as a description of the epidemic in Lombardia (difference in BIC of 28), and weak evidence in favour of a fSEIRDe model in Emilia-Romagna (difference in BIC of 2).

**Table 2:** RMSE and BIC values.

Model	Compartment	Lombardia		Emilia-Romagna	
		RMSE	BIC	RMSE	BIC
fSEIRDr	I	2782	1525	525.5	1303
	R	8281	1723	3289	1636
	D	8417	1726	3463	1646
fSEIRDe	I	3250	1553	520	1301
	R	8001	1717	3291	1636
	D	8163	1721	3464	1646

RMSE and BIC values of the infected (I), Recovered (R) and Dead (D) compartments of fSEIRDr and fSEIRDe models calibrated on data from 24/02/2020 to 24/06/2020.

### Epidemic evolution forecast

In this section we investigate how these models could be used at an early stage of the epidemic evolution, to see how and to what extent they could yield useful information in terms of predicting the epidemic peak of the infected population. To this purpose, we calibrate fSEIRDe and fSEIRDr using the data from 24/02/2020 to 27/03/2020 and then we use the calibrated models to make previsions until 23/06/2020. Observing the results reported in Table 3 we see that the infected peaks are localized quite accurately (maximum three days error). In Emilia-Romagna, both models over-estimate the real peak populations of about [70%–90%]. Conversely, the situation in the Lombardia region is more complicated, and the two models present two different possible evolutions. In this case only fSEIRDr localizes the peak precisely, still overestimating the infected numbers (137%). On the contrary, fSEIRDe finds an epidemic peak 21 days earlier but with a milder underestimate.

**Table 3:** Infected peak days.

	Emilia-Romagna		Lombardia	
	Peak day	Infected	Peak day	Infected
data	13 Apr 2020	13818	04 May 2020	37307
fSEIRDr	11 Apr 2020	24613	03 May 2020	88763
fSEIRDe	10 Apr 2020	26297	13 Apr 2020	30576

Infected Peak days and values for measured data, fSEIRDr and fSEIRDe models calibrated in the period [24/02-27/03].

**Fig 11:**
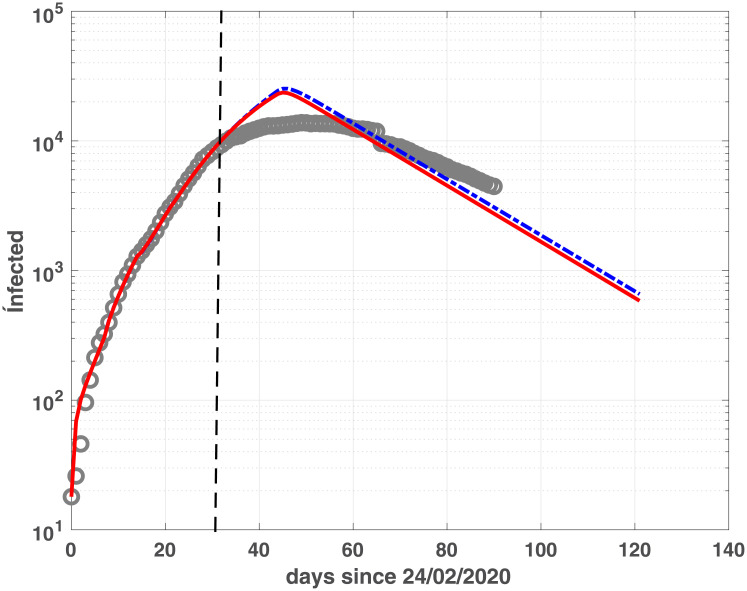
Prevision for infected in Emilia-Romagna. Infected population of fSEIRDe (continuous red line) and fSEIDRr (blue dash-dotted line) calibrated on the first 30 days observed infection data [24/02-27/03], delimited by the vertical black dashed line. Prediction is shown until 30 days after the last measured data (day 24/05). Grey circles represent the infected data in the period [24/02-24/05].

**Fig 12:**
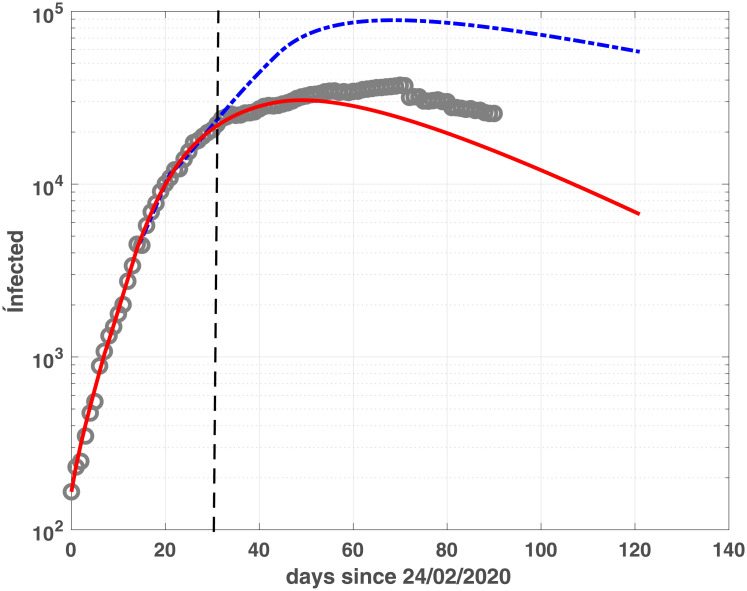
Prevision for infected in Lombardia. Infected population of fSEIRDe (continuous red line) and fSEIDRr (blue dash-dotted line) calibrated on the first 30 days observed infection data [24/02-27/03], delimited by the vertical black dashed line. Prediction is shown until 30 days after the last measured data (day 24/05). Grey circles represent the infected data in the period [24/02-24/05].

The global trend, represented in Figs 11 and 12 for Emilia-Romagna and Lombardia, confirms that fSEIRDr and fSEIRDe can be applied to predict possible epidemic evolutions even from early-stage data. Hence, the different behaviour of the models can be usefully applied to predict different possible future scenarios.

**Fig 13:**
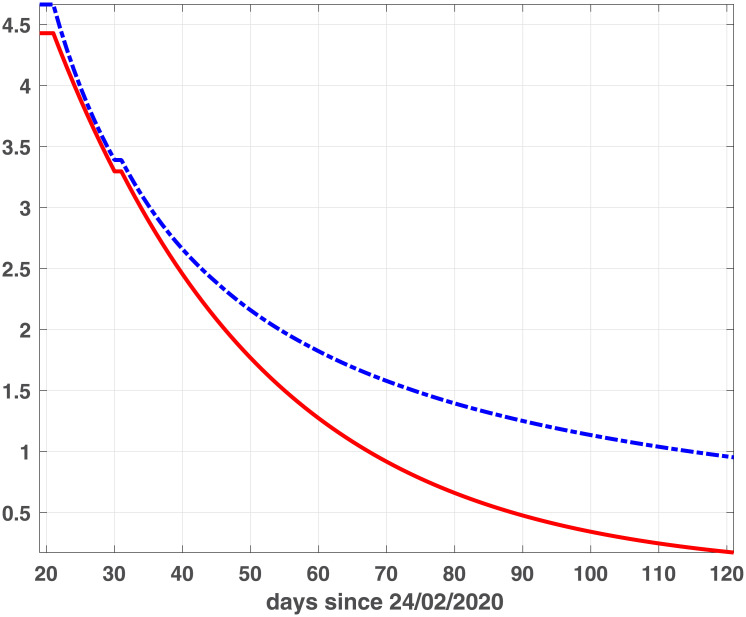
Reproduction function in Emilia-Romagna. ℛ(t) predicted until 24/06/2020 after the calibration of fSEIRDe (red continuous line) and fSEIDRr (blue dash-dotted line) on the data in the period [24/02-27/03].

**Fig 14:**
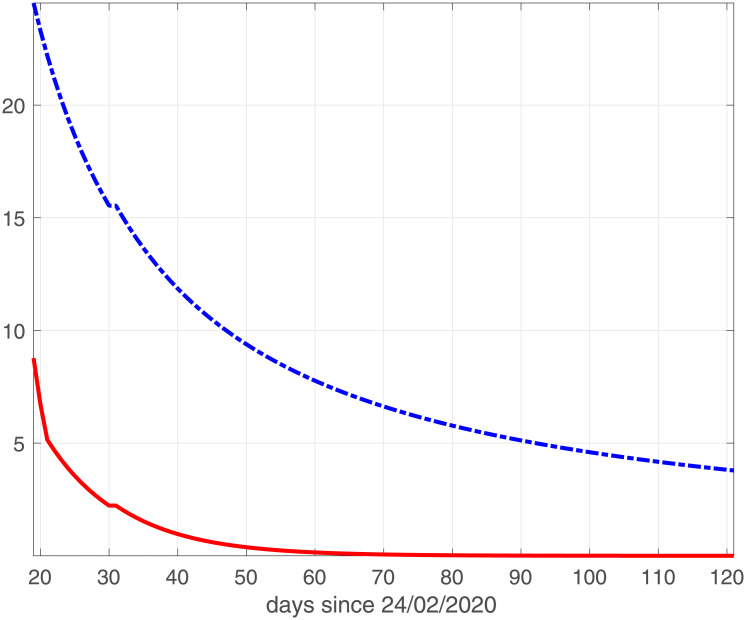
Reproduction function in Lombardia. ℛ(t) predicted until 24/06/2020 after the calibration of fSEIRDe (red continuous line) and fSEIDRr (blue dash-dotted line) on the data in the period [24/02-27/03].

Concerning the basic reproduction functions ℛ(t) in Fig 13 and in Fig 14, we choose to report it from the 20*th* day, to focus our observations on the prediction phase. Regarding the fSEIRDe model we observe that ℛ(t)<1 is achieved at *t* = 41(*d*) (4 April 2020) in Emilia-Romagna and *t* = 69(*d*) (2 May 2020) in Lombardia. The fSEIRDr model never reaches ℛ(t)<1 for Lombardia while for Emilia-Romagna it is reached at *t* = 116(*d*) (20 June 2020). In the case of fSEIRDe model the function ℛ(t) matches the trend of the infected curve, whereas ℛ(t) has too large values for fSEIRDr model. From our observations we believe that two factors cause this misbehaviour: the inaccurate initial value of the exposed population, which should be calibrated, and the evolution of the function *α*(*t*), whose change should be adaptively bounded by a more refined calibration procedure.

## Conclusion

In this paper, we have proposed a forced SEIRD model and investigated two different infection rate functions for the analysis of the COVID-19 outbreak evolution in Italy. In our new formulation, we have partitioned the integration time into sub-intervals, where the model parameters have been adaptively estimated.

The results obtained by fitting the data of two Italian regions, Lombardia and Emilia-Romagna, available from February 24th 2020 until May 24th 2020, show a very good fit to the data. We have then used the model to make predictions by calibrating it only over a short period of about 30 days, and we have compared our prevision with the actual collected data. We believe that the proposed model can be quickly adapted to monitor various infected areas at different epidemic stages. Concerning the Italian epidemic evolution, we are now facing the end of the movement restriction measures, and one crucial challenge is the prediction of potential new epidemic outbreaks, possibly connected to the spread of autumnal influenza. Further studies on forced models will be carried out in this perspective, together with further improvement of the calibration procedure.
